# Cardiac dose evaluation for 3Dimensional conformal partial breast irradiation compared with whole breast irradiation

**DOI:** 10.1120/jacmp.v10i1.2868

**Published:** 2009-01-14

**Authors:** Ashley A. Gale, Anudh K. Jain, Laura A. Vallow, Christopher F. Serago, Steven J. Buskirk, Michael G. Heckman

**Affiliations:** ^1^ Department of Radiation Oncology Mayo Clinic Jacksonville Florida U.S.A.

**Keywords:** partial breast irradiation, cardiac toxicity, 3D conformal external beam irradiation

## Abstract

To compare the radiation dose to normal cardiac tissue for 3Dimensional (3D) conformal external beam partial breast irradiation (PBI) and standard whole breast irradiation (WBI), and examine the effect of tumor bed location.

For 14 patients with left breast tumors randomized on the National Surgical Adjuvant Breast and Bowel Project B‐39 protocol, computer‐generated radiotherapy treatment plans were devised for WBI and PBI. Tumor bed location was designated according to whether more than 50% of the excision cavity was medial or lateral to the nipple line. The volume of heart receiving doses of 2.5, 5, 10, and 20 Gy was calculated for all PBI and WBI plans. Dose to 5% of the heart volume (D5) and mean heart dose were also calculated. The biologically‐equivalent dose (BED) was calculated to account for the different fractionation used in PBI and WBI.

Of the 14 patients, 8 had lateral tumor beds, and 6 had medial tumor beds. The volumes of heart receiving 2.5, 5, 10, and 20 Gy were significantly lower for lateral PBI compared with WBI. For medial PBI, significant cardiac sparing was only seen at a dose of 20 Gy. The difference of D5 values was significant for lateral PBI compared with WBI (p=0.008), but not for medial PBI compared with WBI (p=0.84). The mean dose was also significantly lower for lateral PBI compared with WBI (p=0.008), but not for medial PBI (p=0.16). The results from BED calculations did not change this outcome.

Both 3D conformal PBI and standard WBI can deliver relatively low doses to the heart. For patients with lateralized tumor beds, PBI offers significant cardiac sparing compared with WBI. Patients with medial lesions have relatively similar heart dosimetry with PBI and WBI. 3D conformal PBI is an emerging treatment modality and continued participation on clinical trials is encouraged. Patients with left‐sided lesions and lateralized tumor beds warrant special consideration for PBI, given the significant cardiac dose sparing.

PACS numbers: 87.53.Tf

## I. INTRODUCTION

The estimated incidence of new breast cancer cases in the United States in 2007 is about 180,000, and the estimated number of deaths from breast cancer in 2007 is about 40,000^(1)^. External beam radiotherapy is an essential component of breast conservation therapy in the treatment of breast cancer. Continued refinement of radiotherapy techniques are desired to increase the effectiveness and convenience of treatment and to minimize potential treatment toxicity.

Partial breast irradiation (PBI), as a part of breast conservation therapy, has been proposed as an alternative treatment to conventional whole breast irradiation (WBI) in the management of early‐stage breast cancer.^(^ ^2^ ^,^ ^3^ ^)^ Currently, the National Surgical Adjuvant Breast and Bowel Project (NSABP) and Radiation Therapy Oncology Group (RTOG) are conducting B‐39/RTOG 0413, a randomized phase III study to test the equivalence of PBI and WBI.^(4)^ The primary aim of the NSABP/RTOG protocol is to determine whether the local tumor control probability in the breast is equivalent for the two treatment arms.

Currently only 10% to 40% of women who are candidates for breast conservation actually receive breast conservation therapy.^(4)^ The availability of PBI may result in increased utilization of breast‐conserving therapy. PBI could be a more attractive option because it requires a shorter treatment time than standard WBI; PBI patients are treated twice daily for 10 fractions; as a result, they complete treatment in 5 days as opposed to a 5‐ to 7‐week course for WBI. As part of the B‐39 behavioral and health outcomes studies, it is hypothesized that cosmetic results for PBI will be comparable to those for WBI. It is hypothesized that patients will experience less fatigue with PBI compared to WBI.^(4)^ These factors could make breast conservation a more attractive choice for women with breast cancer.

Breast‐conserving therapy has been established as a safe and effective treatment option for women with early‐stage breast cancer. However, a number of studies suggest that tangential irradiation can cause cardiac injury in some patients.^(^ ^5^ ^–^ ^7^ ^)^ Harris et al.^(6)^ reviewed the medical records of 961 consecutive patients between 1977 and 1994 with breast conservation treatment. They concluded that irradiation of the left breast is associated with an increased risk of coronary artery disease and myocardial infarction compared with irradiation of the right breast. One of the recommendations was to encourage the use of improved radiation techniques to treat left‐sided breast cancer. Borger et al.^(7)^ reported on the incidence of cardiovascular disease in 1,601 patients with breast cancer treated between 1980 and 1993. They concluded that patients treated for left‐sided breast cancer had a higher incidence of cardiovascular disease compared with patients with right‐sided breast cancer. They also urged the use of radiotherapy techniques that minimize the dose to the heart during the treatment of breast cancer. Because the risk of cardiovascular disease has been shown to be higher with left‐sided breast cancer, we selected patients with left‐sided breast cancer for our study.^(^ ^6^ ^–^ ^7^ ^)^

Standard WBI uses tangential beams that include the entirety of the breast. For left‐sided breast cancer, a substantial volume of heart may be included in the tangential fields. PBI techniques, which treat only the tumor bed plus a margin, may potentially reduce the toxic effects on normal heart tissue. However, in order to treat only the region of the tumor bed, typical PBI treatment techniques^(^ ^2^ ^,^ ^8^ ^)^ use multiple noncoplanar beams, the orientations of which are quite different from conventional tangents used for WBI treatment. Thus, while PBI techniques irradiate a smaller region than WBI, it is possible that normal tissues are irradiated with PBI that are not normally irradiated by a WBI technique. It is therefore of interest to compare the normal tissue doses of PBI and WBI.

Comparing cardiac dose between PBI and WBI is of particular interest because cardiac perfusion changes have been seen 6 months after radiation treatment^(^ ^9^ ^–^ ^11^ ^)^ in patients irradiated for breast cancer. These studies found that there was a correlation between the incidence of perfusion defects and the volume of left ventricle irradiated. The study of cardiac dose and its associated risk is important because several studies have shown that the risk of cardiovascular morbidity and mortality increases with WBI after lumpectomy.^(^ ^5^ ^,^ ^6^ ^)^ Additionally, because this low risk patient population can have a long life expectancy, late effects such as cardiac complications may have adequate time to be expressed.

The exact relationship between dose volume information and cardiac injury is still not clear. Published data have even varied as to the quantitative measures of heart dose specifications that have been reported. We looked at mean cardiac dose and dose to 5% of the heart volume (D5) in this study because these parameters have been previously shown to be predictive of coronary heart disease.^(12)^ D5 was also chosen because there is evidence that a small volume of heart exposed to large doses of radiation can result in significant cardiac complications.^(^ ^11^ ^,^ ^13^ ^)^

The difference in fraction size is another factor that should be considered when comparing dose parameters between PBI and WBI. The treatment regimens used in the protocol are a daily dose of 1.8 to 2.0 Gy for 25 to 35 fractions for WBI, compared with a PBI external beam dose of 3.85 Gy per fraction for 10 fractions. Cosset et al.^(14)^ found that the incidence of pericarditis increased when the dose per fraction was increased. The heart is regarded as a late‐responding tissue with sensitivity to fraction size, so this should be considered when evaluating heart dose and risk for late effects. In our study we used the biologically‐equivalent dose (BED) to account for the different fractionation used for PBI and WBI.

The B‐39 protocol hypothesizes that one of the benefits of PBI compared with WBI is the reduced toxicity to normal structures, including the heart. Reduced toxicity is expected with PBI based on the assumption that the dose to normal tissues is reduced. We postulate that the dose to normal heart tissue is dependent on patient anatomy, and that the location of the tumor bed in the breast may also be an important factor. The purpose of this study was to compare the dose to cardiac tissue for 3D conformal external beam PBI and standard WBI. The effect of tumor bed location on cardiac dose was also evaluated. The linear‐quadratic model was used to calculate the BED to account for the difference in fractionation between the two regimens.^(15)^

## II. MATERIALS AND METHODS

This study was approved by the Mayo Clinic Institutional Review Board. From October 2005 to May 2007, 14 sequentially treated patients with left breast tumors who were randomly assigned on the NSABP B‐39 protocol at Mayo Clinic Jacksonville were selected for a comparison of WBI and 3D conformal PBI. To be eligible for the B‐39 protocol the patients were women at least 18 years old who had stage 0, I, or II breast cancer, with tumor size of 3 cm or less. Patients with suspicious ipsilateral or contralateral axillary, supraclavicular, infraclavicular, or internal mammary nodes were excluded unless histologic evidence proved these nodes were negative for tumor. For the purpose of this study, both WBI and PBI treatment plans were generated for each patient, regardless of how the patient was randomized for the protocol. This design eliminated anatomic and tissue contouring as potential sources of bias. Patients were categorized as having either a medial or a lateral tumor. Tumor bed location was designated according to whether more than 50% of the excision cavity was either medial or lateral to the nipple line.

A computed tomography (CT) simulation was performed with the patient supine in an immobilization mold with arms above the head. The clinical breast borders were outlined with radiopaque markers by the radiation oncologist. A CT scan slice thickness of 3 mm was used. The CT data set was then transferred to the Pinnacle treatment planning workstation (Philips Medical Systems, N.A., Bothell, Washington). All required structures were contoured by the radiation oncologist and medical physicist to comply with dose volume limitations of the B‐39 protocol, listed in Table 1. A full description of the dose constraints found in Table 1 is given in the B‐39 protocol.^(4)^ The excision cavity was contoured on the basis of CT visualization. The heart volume was contoured starting just below the branches of the pulmonary trunk to the most inferior part of the heart near the diaphragm. All mediastinal tissue, including the great vessels, was included in the heart contour.

**Table 1:** NSABP B‐39/RTOG 0413 Dose Constraints for Left‐Sided External Beam PBI.

	*Objective*
PTVEVAL	>90% should receive 3465 cGy
Max Dose	<4620cGy
Ipsilateral Breast	<60% should receive ≥1925cGy
Ipsilateral Breast	<35% should receive >3850cGy
Contralateral Breast	Max point dose <115.5 cGy
Ipsilateral Lung	<15% can receive 1155 cGy
Contralateral Lung	<15% can receive 192.5 cGy
Heart (Left)	<40% can receive 192.5 cGy
Thyroid	Max point dose <115.5cGy

We used the B‐39 protocol definition of PTV. The definition of PTV in previous studies has not been consistent, ranging from lumpectomy plus 1.5–2.0 cm to 2.0–2.5 cm. Protocol B‐39 defines the clinical target volume (CTV) as lumpectomy plus 1.5 cm, not including chest wall and pectoralis muscles, and a limit of 5 mm from the skin surface. The PTV is defined as CTV plus 1.0 cm. An additional region of interest defined in B39 is the PTV for Evaluation (PTV_EVAL). This structure is used for dose volume histogram constraints and analysis. The PTV_EVAL is defined as the PTV excluding the first 5 mm of tissue under the skin, and excluding the chest wall, pectoralis muscles, and lung.

**Figure 1:**
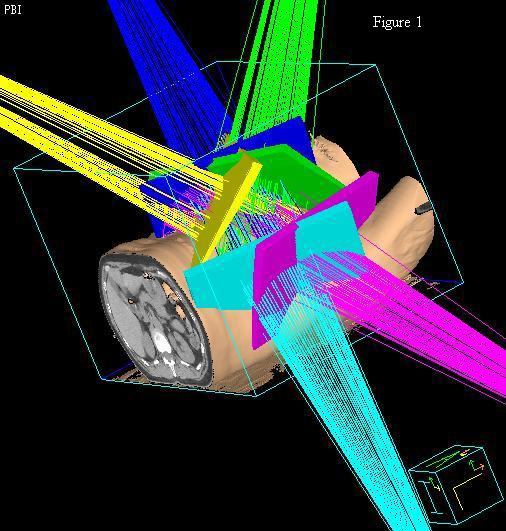
Representative example of the beam arrangement for a typical PBI treatment.

All treatment plans were done with a 6 MV photon beam and patients were treated on a Varian 23EX or 23IX linear accelerator (Varian Medical Systems, Palo Alto, California). PBI beam arrangement was guided by the technique that has been adopted by the B39 protocol, described by Baglan et al.^(2)^ This multiple noncoplanar photon field technique was found to be superior to mixed‐modality plans.^(8)^ Typically, our study used 5 noncoplanar beams for PBI plans (Fig. 1). The same planner generated all the PBI plans in order to avoid inter‐planner variability. The beam arrangement was tailored to patient geometry in order to comply with the required dose volume constraints of the protocol. The beam arrangement was also chosen in order to ensure that no beam was directed toward critical structures.^(4)^ The beams consisted of a right anterior superior to inferior oblique, right anterior lateral, right anterior inferior to superior oblique, left posterior superior to inferior oblique, and left posterior inferior to superior oblique. The 3 medial tangents approximated a medial breast tangent with 10–20 degree steeper gantry angle than whole breast tangents and couch angles of 15–70 degrees. The posterior fields used couch angles between 10 and 20 degrees and shallower gantry angles than traditional tangents. More weighting was typically given to the medial tangents. The isocenter was placed at the center of the PTV. The prescription dose was 38.5 Gy in 10 fractions to the isocenter. Either wedges or the field‐in‐field technique was used when appropriate. Heterogeneity correction was used for all plans.

**Figure 2:**
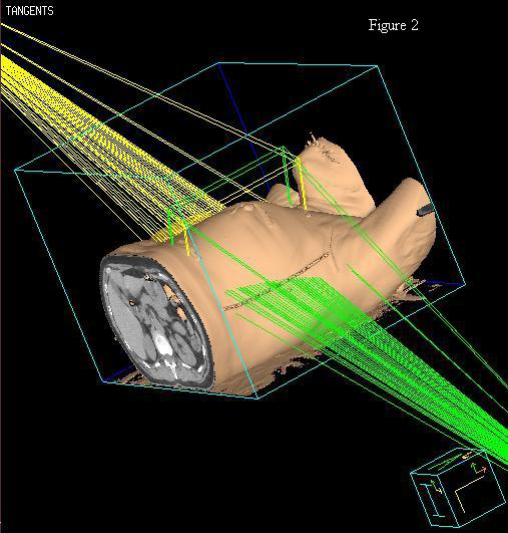
Representative example of the beam arrangement for a typical WBI treatment.

For WBI planning, two tangent fields were used (Fig. 2). The tangents were opposed fields encompassing the clinical breast volume, approximating a half beam block. The dose was normalized to a point two thirds the perpendicular distance from the skin to the posterior border of the field at mid‐separation on the isocenter slice. The radiation oncologist determined the clinical breast volume treated by the tangents. A dose of 50 Gy in 25 fractions was used. The field‐in‐field technique was used for all WBI plans. Either three or four subfields were used to improve dosimetric coverage.

For each WBI plan and PBI plan, the volume of heart receiving 2.5 Gy, 5 Gy, 10 Gy, and 20 Gy was obtained from the treatment planning system (Pinnacle). This was expressed as a percentage of the total heart volume (V2.5, V5, V10, V20) for each patient. D5 and mean heart dose were also recorded for all plans.

For comparison of doses to account for fractionation, the linear quadratic equation, shown below, was used to calculate BED (Gy3) (16) for D5 and mean heart dose:

(1) BED(GY3)=nd[1+d/(α/β)]

where n is the number of fractions, d is the dose per fraction and α/β is the alphabeta ratio. Since the primary clinical effects from heart radiation are late effects (cardiac perfusion changes, coronary artery disease), an alpha/beta ratio of 3 was chosen.^(16)^ The calculation shown below as an example is for a single patient with a total D5 value of 8.37 Gy for PBI treatment and a total D5 value of 5.68 Gy for WBI treatment:

PBI:
BED(Gy3)=Number of fractions(10)×dose per fraction(0.837Gy)[1+dose per fraction(0.837Gy)/alpha beta(3)]=10.71Gy3
WBI:
BED(Gy3)=Number of fractions(25)×dose per fraction(0.227Gy)[1+dose per fraction(0.227Gy)/alpha beta(3)]=6.11Gy3

Thus, using an alpha/beta ratio of 3, 6.11 Gy3 in 25 fractions can be considered biologically‐equivalent to 10.71 Gy3 in 10 fractions. For a biologically‐equivalent comparison of dose distribution for this patient, 6.11 Gy3 in the WBI plan was compared to 10.71 Gy3 in the PBI plan. These calculations were done for each patient in order to compare biologically‐equivalent D5 values for WBI and PBI. The mean lung dose for the WBI and PBI were also converted to Gy3 using the BED formula above and compared.

Numerical variables were summarized with the sample mean, median 25th percentile, and median 75th percentile. Categorical variables were summarized with number and percentage. A Wilcoxon signed rank test was used to compare outcomes between PBI and WBI treatments, separately for medial and lateral tumors.^(17)^ A Wilcoxon rank sum test was used to investigate whether the associations between dose outcomes and treatment differed between medial and lateral tumors. P‐values ≤0.05 resulting from the aforementioned tests were considered statistically significant. No adjustments for multiple testing were made in these exploratory analyses.

## III. RESULTS

The 14 patients evaluated were representative of the low‐risk breast cancer patients eligible for the B‐39 trial at the time of randomization. The patients chosen for this study all had left‐sided breast cancer. Patient characteristics are listed in Table 2. Of the 14 patients, 8 had lateral tumor beds and 6 had medial tumor beds. PBI and WBI plans were successfully performed on all 14 patients and were within protocol guidelines. For 13 of the patients, 5 non‐coplanar beams were used for the PBI plans and only 1 used 4 non‐coplanar beams.

**Table 2:** Patient characteristics

Variable	Summary (N=14)
Age	Mean: 62 (range: 26–86)
Tumor size (cm)	Mean: 1.1 (range: 0.2–1.9)
Histology	
Infiltrating ductal carcinoma	10 (71%)
Ductal carcinoma *in situ*	3 (21%)
Infiltrating lobular carcinoma	1 (7%)
Tumor location	
Medial	6 (43%)
Lateral	8 (57%)
Number of beams	
4	1 (7%)
5	13 (93%)

**Figure 3:**
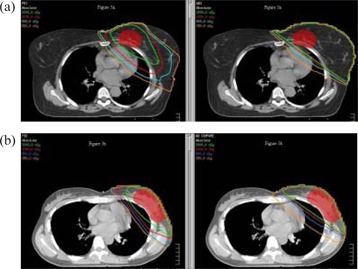
Sample isodose lines for a patient with a medial tumor bed (a) and a patient with a lateral tumor bed (b). In each pair of images, the partial breast irradiation plans are on the left, and the whole breast irradiation plans are on the right. The planning target volume is shown in red.

**Figure 4:**
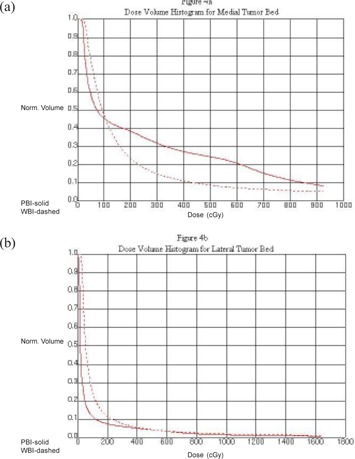
Sample dose volume histograms for a patient with a medial tumor bed (a) and a patient with a lateral tumor bed (b). In both figures, the solid line represents partial breast irradiation and the dashed line represents whole breast irradiation.

Table 3 shows the percentage of total heart volume averaged for all patients at doses of 2.5, 5, 10, and 20 Gy for medial and lateral tumor beds. For patients with lateral tumor beds, the volume of heart was significantly lower with PBI than with WBI for all doses evaluated. However, cardiac sparing was not as notable for patients with medial lesions. For these patients, the volume of heart was only significantly lower with PBI than WBI for 20 Gy. Figs. 3(a) and 3(b) show a representative comparison of a PBI and WBI treatment plan for a medial tumor bed and a lateral tumor bed, respectively. A representative dose volume histogram comparing PBI and WBI is shown in Fig. 4(a) for a medial tumor bed and Fig. 4(b) for a lateral tumor bed.

**Table 3:** Percentage volume of heart exposed to 2.5 Gy, 5 Gy, 10 Gy, and 20 Gy for whole breast irradiation and partial breast irradiation

*Irradiation dose*	*Volume of heart exposed to irradiation (%)*		*P‐value*
	*PBI*	*WBI*	
	*Medial Tumors*		
2.5 Gy	8.1 (3.4, 21.8)	12.2 (7.2, 17.5)	1.00
5 Gy	4.5 (1.4, 12.9)	4.8 (2.3, 7.8)	0.84
10 Gy	1.8 (0.4, 3.3)	2.2 (0.9, 4.2)	0.84
20 Gy	0.4 (0.1, 0.5)	1.0 (0.3, 2.5)	0.031
	*Lateral Tumors*		
2.5 Gy	0.2 (0.2, 5.4)	14.9 (8.2, 18.1)	0.008
5 Gy	0.0 (0.0, 3.0)	3.6 (1.4, 5.0)	0.008
10 Gy	0.0 (0.0, 1.7)	1.5 (0.5, 2.4)	0.008
20 Gy	0.0 (0.0, 0.1)	0.6 (0.1, 1.2)	0.023

The sample mean (median 25^th^ percentile, median 75^th^ percentile) is given. P‐values result from a Wilcoxon signed rank test comparing PBI vs WBI.

In this study, both 3D conformal PBI and standard WBI plans achieved relatively low doses to the heart. The dosimetric results are shown in Table 4 for medial and lateral tumor beds. This table demonstrates that patients with lateral tumor beds treated with PBI had more heart sparing, and patients with medial lesions treated with PBI or who received WBI had a similar heart dose. The difference of D5 values was significant for lateral PBI compared with WBI (p=0.008), but not for medial PBI compared with WBI (p=0.84). The mean dose was also significantly lower for lateral PBI compared with WBI (p=0.008), but not for medial PBI (p=0.16). Taking BED into account did not change the statistical significance of the results (See Table 4).

**Table 4:** Mean dose, BED mean dose, D5, and BED D5 for whole breast irradiation and partial breast irradiation

*Variable*	*PBI Medial tumors*	*WBI*	*P‐value*
Mean dose (Gy)	1.1 (0.5, 2.0)	1.6 (1.0, 2.4)	0.16
BED mean dose (Gy3)	1.1 (0.6, 2.1)	1.6 (1.0, 2.5)	0.31
D5 (Gy)	4.4 (2.2, 7.5)	4.8 (3.2, 8.4)	0.84
BED D5 (Gy3)	5.1 (2.4, 9.5)	5.2 (3.4, 9.3)	1.00
	*Lateral tumors*		
Mean dose (Gy)	0.33(0.27,0.75)	1.47(1.08,2.05)	0.008
BED mean dose (Gy3)	0.34(0.27, 0.77)	1.50(1.10,2.11)	0.008
D5 (Gy)	1.1 (1.0, 2.7)	4.1 (3.2, 5.1)	0.008
BED D5 (Gy3)	1.1 (1.0, 3.0)	4.4 (3.3, 5.4)	0.008

The sample mean (median 25^th^ percentile, median 75^th^ percentile) is given. P‐values result from a Wilcoxon signed rank test comparing PBI vs WBI. BED is defined as Biologically Equivalent Dose. D5 is defined as dose to 5% of the total heart volume.

## IV. DISCUSSION

In this study, we dosimetrically compared 3D conformal PBI and WBI for the same 14 patients. For patients with lateral tumor beds, PBI treatments resulted in a significantly smaller volume of irradiated heart for all doses evaluated as well as a significant reduction in D5 and mean heart dose.

Because there is a smaller volume of tissue treated with PBI, it is conceivable that an even larger fraction size may be used, thus allowing for shorter overall treatment time. The heart is a structure that is sensitive to fractionation with an alpha/beta ratio of 3 and this must be considered with any hypofractionation regimen for breast cancer.^(^ ^15^ ^–^ ^16^ ^,^ ^18^ ^–^ ^19^ ^)^ BED values were used in this study so that the dose received using a different number of fractions could be compared. The biological disadvantages of hypofractionation for normal tissue did not change the results significantly for mean dose or D5.

A previous study on this subject, Hiatt et al.,^(20)^ reported a comparison of cardiac dose associated with WBI and PBI for left‐sided breast cancer. They specifically selected 9 patients whose anatomy was unfavorable in relation to the proximity of the heart to the high radiation dose volume. They concluded that PBI techniques more effectively reduce cardiac dose than WBI techniques. They added that the relative sparing of cardiac tissue is reduced when the tumor bed is in the medial portion of the breast. Our experience shows that the sparing of cardiac tissue is statistically significant when the tumor bed is located laterally in the breast. We found this also to be true when fractionation was taken into account. Additionally, our study was not limited to patients with unfavorable anatomy, and therefore our results may be applicable to a general patient population.

There have been mixed results regarding cardiac dose‐response relationships in recent studies. One study found a statistically significant elevated risk of coronary heart disease for patients who received a mean heart dose of 2.8 Gy and D5 value of 12.9 Gy.^(12)^ Taylor et al.^(21)^ suggested that the dose to different cardiac structures may be a more important predictor of cardiac morbidity and mortality than the volume of heart irradiated. Coronary artery doses have been reported, but it has not been determined whether a high dose to this area has clinical consequences.^(^ ^21^ ^–^ ^23^ ^)^ To determine the probability of cardiac risk from the doses and volumes quantified in our study, further investigation on the relationships between cardiac dose and morbidity and mortality is needed.

Various approaches have been taken to reduce the dose to organs at risk during breast irradiation. These methods include 3D dose planning, inclusion of an electron field, proton therapy, intensity‐modulated radiation therapy (IMRT), and respiratory gating. Kozak et al.^(24)^ showed that protons used in PBI reduce the dose delivered to the heart compared with mixed‐modality 3D PBI. IMRT has been shown to improve PBI.^(25)^ Moreover, deep inspiration breath‐hold has the potential to significantly reduce heart dose for left‐sided breast cancer treatment using tangential fields.^(^ ^26^ ^–^ ^29^ ^)^ All these methods have proven that it is possible to reduce the dose to the heart to relatively low levels without compromising PTV coverage. Our study shows that 3D conformal photon PBI is also a method that can be used to reduce dose to the heart for left‐sided lateral tumor beds.

## V. CONCLUSIONS

Both 3D conformal PBI and standard WBI can be delivered with relatively low doses to the heart. For patients with left‐sided lateralized tumor beds, we found that 3D conformal PBI offers significant sparing of cardiac tissue compared with standard WBI. However, patients with left‐sided medial lesions have relatively similar heart dosimetry with 3D conformal PBI and WBI. Longer follow‐up in a larger number of patients is needed to increase the power to detect trends.

Even with modern techniques it may be unavoidable to irradiate part of the heart. Continued improvements of techniques which reduce the dose to the heart are recommended. The decision to use 3D conformal PBI is a matter of continued debate, and the authors encourage participation on clinical trials. The selection of patients should take into account many factors such as recurrence risk, age, comorbidities, and resource availability. However, patients with left‐sided tumors and lateralized tumor beds deserve special consideration for this technique due to the significant cardiac sparing demonstrated in this study.
